# RAD51 protein is a predictor of chemosensitivity and survival prognosis in patients with advanced high-grade serous ovarian cancer undergoing neoadjuvant chemotherapy

**DOI:** 10.3389/fonc.2025.1548889

**Published:** 2025-10-22

**Authors:** Hui Liu, Yufang Xia, Yunqing Chen, Huiyang Qi, Huixiang Ji, Shujun Ji, Yanhui Lou

**Affiliations:** ^1^ Department of Gynecology, the Affiliated Hospital of Qingdao University, Qingdao, China; ^2^ Department of Reproductive Medicine, Linyi People’s Hospital, Linyi, Shandong, China; ^3^ Department of Pathology, Affiliated Hospital of Qingdao University, Qingdao, Shandong, China; ^4^ Department of Obstetrics & Gynecology, Rizhao People’s Hospital, Rizhao, China

**Keywords:** RAD51 protein, KELIM score, high-grade serous ovarian cancer, neoadjuvant chemotherapy, chemotherapy sensitivity

## Abstract

**Objective:**

The primary aim of this study is to investigate the relationship between the expression of the homologous recombination protein RAD51 and the CA125 elimination rate constant K (KELIM) score in the context of sensitivity to neoadjuvant chemotherapy (NACT) in patients with advanced high-grade serous ovarian cancer. Additionally, we explore the potential of RAD51 expression and the KELIM score as biomarkers of chemotherapy sensitivity.

**Methods:**

We selected a cohort of 43 patients with advanced high-grade ovarian carcinoma who underwent intermediate tumor cytoreductive surgery (IDS) following NACT between January 2017 and December 2019. Pathological tissue samples were collected from pre-chemotherapy and post-IDS ovarian cancer tissues, as well as from normal ovarian tissues of 12 control subjects. Immunohistochemistry was used to evaluate RAD51 protein expression. Concurrently, the KELIM score was calculated for NACT patients. Progression-free survival (PFS) and overall survival (OS) were monitored, and the correlation between RAD51 expression, chemotherapy sensitivity, and survival outcomes was assessed. Furthermore, we analyzed the combined prognostic value of RAD51 expression and the KELIM score in predicting NACT sensitivity and prognosis in advanced high-grade serous ovarian cancer.

**Results:**

The expression rate of RAD51 protein in ovarian cancer tissues was significantly higher compared to normal ovarian tissues (95.3%VS 16.7%, P < 0.05). High RAD51 expression was significantly negatively correlated with the KELIM score and NACT sensitivity. Both RAD51 expression and the KELIM score were associated with the recurrence of platinum resistance after surgery. Patients with high RAD51 expression exhibited a higher recurrence rate of platinum resistance compared to those with low RAD51 expression. Similarly, patients with a KELIM score < 1 had a statistically significantly higher recurrence rate of platinum resistance than those with a KELIM score ≥ 1 (P < 0.001). There was no significant statistical difference between the AUC of RAD51 expression for predicting platinum-resistant recurrence and that of the KELIM score (P> 0.05). The AUC of the combination of RAD51 expression and the KELIM score showed an increasing trend compared with the KELIM score and RAD51 expression respectively, yet there was no statistical difference among the three. High RAD51 expression was associated with lower PFS and OS, indicating a poorer survival prognosis.

**Conclusion:**

RAD51 protein expression is closely related to the sensitivity of neoadjuvant chemotherapy in advanced high-grade serous ovarian cancer. RAD51 protein expression offers a valuable tool for predicting chemotherapy sensitivity, platinum resistance recurrence, and survival outcomes in patients with advanced epithelial ovarian cancer.

## 1 Introduction

High-grade serous ovarian cancer (HGSOC), the most common form of epithelial ovarian cancer (EOC), is notable for its high aggressiveness and lethality (1). The management of ovarian cancer relies heavily on effective cytoreductive surgery and platinum-based chemotherapy. In recent years, neoadjuvant chemotherapy (NACT) has become a crucial approach for patients with advanced-stage disease who are not candidates for primary debulking surgery (PDS). NACT can simplify surgical procedures, facilitate the completion of interval debulking surgery (IDS), and reduce perioperative morbidity and mortality. However, the impact of NACT on overall survival and progression-free survival, as well as its potential to induce platinum resistance, remains a topic of ongoing debate (2–4). Furthermore, the high heterogeneity of ovarian cancer results in some tumors exhibiting primary platinum resistance, which not only leads to a poor response to NACT but may also worsen platinum resistance due to the increased tumor burden prior to chemotherapy (5).

Platinum resistance poses a significant challenge for patients undergoing NACT-IDS and has been identified as an independent risk factor for the recurrence of platinum resistance following initial surgery in advanced EOC (6, 7). Therefore, identifying patients at risk for drug resistance prior to treatment initiation is essential for optimizing surgical and chemotherapy strategies. Recent studies have emphasized the utility of the modeled CA125 elimination rate constant K (KELIM) in predicting chemotherapy sensitivity and survival outcomes in ovarian cancer patients after NACT and PDS (8, 9). A higher KELIM score is indicative of improved clearance and greater chemotherapy sensitivity. Nonetheless, since the KELIM score requires multiple CA125 measurements during chemotherapy for calculation, it cannot be used as a pre-treatment screening tool to identify patients with primary platinum resistance, highlighting a gap in pre-chemotherapy predictive capabilities.

Platinum-based chemotherapy agents exert their effects by binding to tumor cell DNA, causing chromosomal double-strand breaks (DSBs) that impair DNA repair, inhibit cell proliferation, and ultimately lead to cell death. Homologous recombination (HR) is a vital repair pathway for DSBs induced by platinum agents, playing a crucial role in maintaining genomic stability and diversity. RAD51 protein is a key component of HR and DNA double-strand break repair, functioning alongside its paralogs (XRCC2, XRCC3, Rad51L1, Rad51L2, and Rad51L3) in HR-mediated repair mechanisms (10). Dysregulation of RAD51 expression and function can disrupt DNA repair processes and contribute to genomic instability. Previous studies have shown that overexpression of RAD51 in various cancers, including esophageal adenocarcinoma, colon cancer, breast cancer, and ovarian cancer, is associated with drug resistance, immune evasion, and a poor prognosis (11, 12). The present study aims to assess RAD51 protein expression in tumor tissues of patients with advanced HGSOC, analyze its correlation with NACT chemotherapy sensitivity and prognosis, and evaluate the potential of RAD51 as a predictive marker for chemotherapy sensitivity in NACT. Additionally, the combined prognostic value of RAD51 expression and the KELIM score will be explored to enhance the prediction of patient outcomes.

## 2 Information and methodology

### 2.1 Clinical data

A total of 43 patients with advanced high-grade serous ovarian carcinoma (HGSOC) who underwent neoadjuvant chemotherapy (NACT) followed by interval debulking surgery (IDS) at the Department of Gynecology, Affiliated Hospital of Qingdao University, between January 2017 and December 2019, were enrolled in this study. Pathological tissue samples were collected from all patients prior to chemotherapy, and an additional 12 normal ovarian tissue samples were obtained for comparison. The inclusion criteria were as follows: patients aged 18 years or older, with a diagnosis of advanced-stage (III–IV) HGSOC, without any comorbidities that could potentially affect the immunohistochemical results, no significant cardiovascular or cerebrovascular diseases, at least three CA125 measurements taken within 100 days of chemotherapy initiation, and completion of NACT followed by IDS with either R1 or R0 resection. Postoperative pathological diagnosis was confirmed for all patients, who received 2–3 cycles of platinum-based NACT followed by 6–8 cycles of platinum-based chemotherapy after IDS. Recurrence within 6 months of treatment completion was considered indicative of platinum resistance, while recurrence after 6 months or later was classified as platinum-sensitive. The follow-up endpoint was defined as either patient death or the completion of a 5-year observation period.

### 2.2 SP Immunohistochemistry was utilized to assess the expression of RAD51 protein in advanced HGSOC tissues (pre-NACT tumor biopsy samples and post-IDS tumor tissues)

The main reagents used in this study were the RAD51 recombinant rabbit monoclonal antibody (primary antibody), purchased from Hua an Biotechnology Co., Ltd., which was used at a working concentration of 1:1000. The procedure comprised the following steps: paraffin sectioning and baking, deparaffinization, antigen retrieval, elimination of endogenous peroxidase activity with 3% H2O2, incubation with the primary antibody (diluted 1:1000) at 37°C for 2–3 hours, washing with PBS, incubation with the secondary antibody at room temperature for 40 minutes, color development, hematoxylin counterstaining, alcohol dehydration, neutral gum mounting, and finally, microscopic observation.

For the determination of results, RAD51 protein expression was considered positive when yellowish to tan granules were observed in the nucleus and/or cytoplasm, and negative when brown granules were absent or the staining matched the background color of the cytoplasm. For each tissue section, five high-power fields were randomly selected, and 100 cells were counted per field. Both the staining intensity and the percentage of positive cells were scored. The staining intensity was rated as follows: 0 points for no coloration, 1 point for light yellow, 2 points for brownish yellow, and 3 points for tan. The number of positive cells was scored based on the following criteria: 0 points for <5% positive cells, 1 point for 5-25%, 2 points for 26-50%, 3 points for 51-75%, and 4 points for ≥76% positive cells. The final score was calculated by multiplying the staining intensity score and the percentage of positive cells score. A score of 0 indicated negative expression, while scores ranging from 1 to 12 indicated positive expression. Scores between 1 and 7 were categorized as low expression, and scores of 8 or higher were considered high expression.

### 2.3 KELIM score calculation

The KELIM score was calculated by inputting at least three CA125 values obtained within the first 100 days of chemotherapy into the online calculation tool at https://www.biomarker-kinetics.org/. A KELIM score ≥ 1 indicated sensitivity to chemotherapy, while a score < 1 indicated resistance.

### 2.4 Statistical methods

Statistical analyses were performed using SPSS 27.0 software. The chi-square test was used to compare rates between groups. The receiver operating characteristic (ROC) curve was employed to assess the predictive efficacy of RAD51 expression and KELIM score for chemotherapy sensitivity in HGSOC. Survival analysis was conducted using the Kaplan-Meier method, with P < 0.05 considered statistically significant.

## 3 Results

### 3.1 Assessment of RAD51 protein expression levels in different types of ovarian tumor tissues

**Figure 1:**
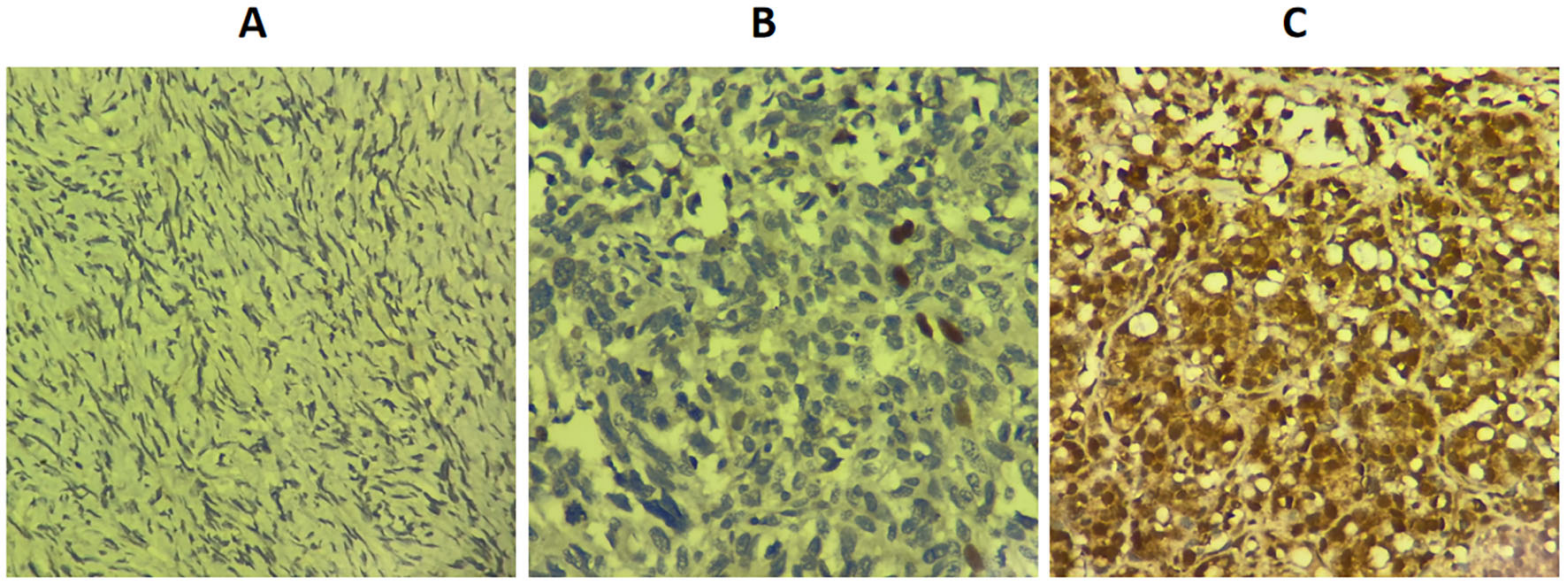
Expression level of RAD51 protein in different ovarian tissues (SP × 400). **(A)** Normal ovarian tissue. **(B)** Low RAD51 expression in high-grade serous ovarian cancer. **(C)** High RAD51 expression in high-grade serous ovarian cancer.

Immunohistochemical analysis demonstrated that RAD51 protein was expressed in both the nucleus and cytoplasm of HGSOC tissues, with a predominant nuclear localization (**Figure 1**). The positive expression rate of RAD51 in HGSOC tissues was markedly higher than that observed in normal ovarian tissue, with rates of 95.3% and 16.7%, respectively. This difference was statistically significant (P < 0.05) (**Table 1**). Out of the 43 cases of HGSOC, 16 exhibited high RAD51 expression, whereas 27 displayed low expression. It is noteworthy that, among the 27 cases with low expression, 2 were completely negative for RAD51 expression. As shown in **Table 2**, there was no statistically significant correlation between RAD51 protein expression and various factors, including age, FIGO stage (III, IV), lymph node metastasis, ascites, or the mean CA125 value prior to the initiation of the first chemotherapy cycle (P > 0.05).

**Table 1:** Expression of RAD51 protein in different types of ovarian tissues.

Types of tissues	RAD51 protein			Positive expression rate (%)	χ^2^	P
	n	masculine	feminine			
Constituencies						
High-grade serous ovarian cancer	43	41	2	95.3	34.050	<0.001
Normal ovarian tissue	12	2	10	16.7		

**Table 2:** Correlation between RAD51 protein expression and clinicopathological characteristics in patients.

Characteristics	RAD51 protein		χ^2^	P
	High expression (n)	Low expression (n)		
Age (years)				
≥ 50	12	23	0.688	0.407
< 50	4	4		
FIGO stage				
III	13	19	0.625	0.429
IV	3	8		
Lymph node metastases				
Yes	11	11	3.154	0.076
No	5	16		
Ascites				
Positive	12	13	2.976	0.084
Negative	4	14		
First CA125 mean before chemotherapy				
≥ 1208	3	7	0.290	0.590
< 1208	13	20		

### 3.2 Relationship between RAD51 protein expression and neoadjuvant chemotherapy sensitivity in patients

Among the 16 patients with high RAD51 protein expression, 12 patients (representing 75.0%, or 12 out of 16) had a KELIM score of less than 1, while the remaining patients had a KELIM score of 1 or higher. Conversely, among the 27 patients with low RAD51 protein expression, 17 patients (63.0%, or 17 out of 27) had a KELIM score of 1 or higher, and the rest had a KELIM score of less than 1. The difference in KELIM scores between the two groups was statistically significant (P = 0.016), as illustrated in **Table 3**. These findings suggest that RAD51 protein expression in ovarian cancer tissues is closely related to the KELIM score and chemotherapy sensitivity. High RAD51 expression is correlated with low KELIM scores and reduced sensitivity to neoadjuvant chemotherapy, whereas low RAD51 expression is associated with high KELIM scores and increased sensitivity to neoadjuvant chemotherapy.

**Table 3:** Relationship between RAD51 protein expression and KELIM score.

Variable	RAD51 protein		χ^2^	P
	High expression n (%)	Low expression n (%)		
KELIM score					
≥ 1	4 (25.0%)	17 (63.0%)	5.795	0.016
< 1	12 (75.0%)	10 (37.0%)		

### 3.3 Association between RAD51 protein expression, KELIM score, and platinum resistance recurrence in patients

In this study, we observed that 19 patients experienced recurrence of platinum resistance. Among them, 13 patients (68.4%, or 13 out of 19) had high RAD51 protein expression, while 6 patients (31.6%, or 6 out of 19 had low expression. In contrast, among the patients with platinum-sensitive recurrence, 87.5% (21 out of 24) exhibited low RAD51 protein expression, with only 3 cases (12.5%, or 3 out of 24) showing high RAD51 expression. The difference between these two groups was statistically significant (P < 0.001), indicating a strong correlation between high RAD51 expression and the recurrence of platinum resistance.

Furthermore, among patients with platinum-resistant recurrence, 78.9% (15 out of 19) had a KELIM score of less than 1, which was significantly higher than the 21.1% (4 out of 19) with a KELIM score of 1 or higher. This suggests that patients with lower chemotherapy sensitivity are more prone to developing platinum resistance after surgery, and the difference was statistically significant (P < 0.05), as illustrated in **Table 4**.

**Table 4:** Relationship between RAD51 protein and KELIM score and platinum resistance relapse.

Variables	PFS		χ^2^	P
	≥ 6 n (%)	< 6 n (%)		
RAD51 expression					
High expression	3 (12.5%)	13 (68.4%)	14.194	<0.001
Low expression	21 (87.5%)	6 (31.6%)		
KELIM score					
≥ 1	17 (70.8%)	4 (21.1%)	10.518	0.001
< 1	7 (29.2%)	15 (78.9%)		

**Figure 2:**
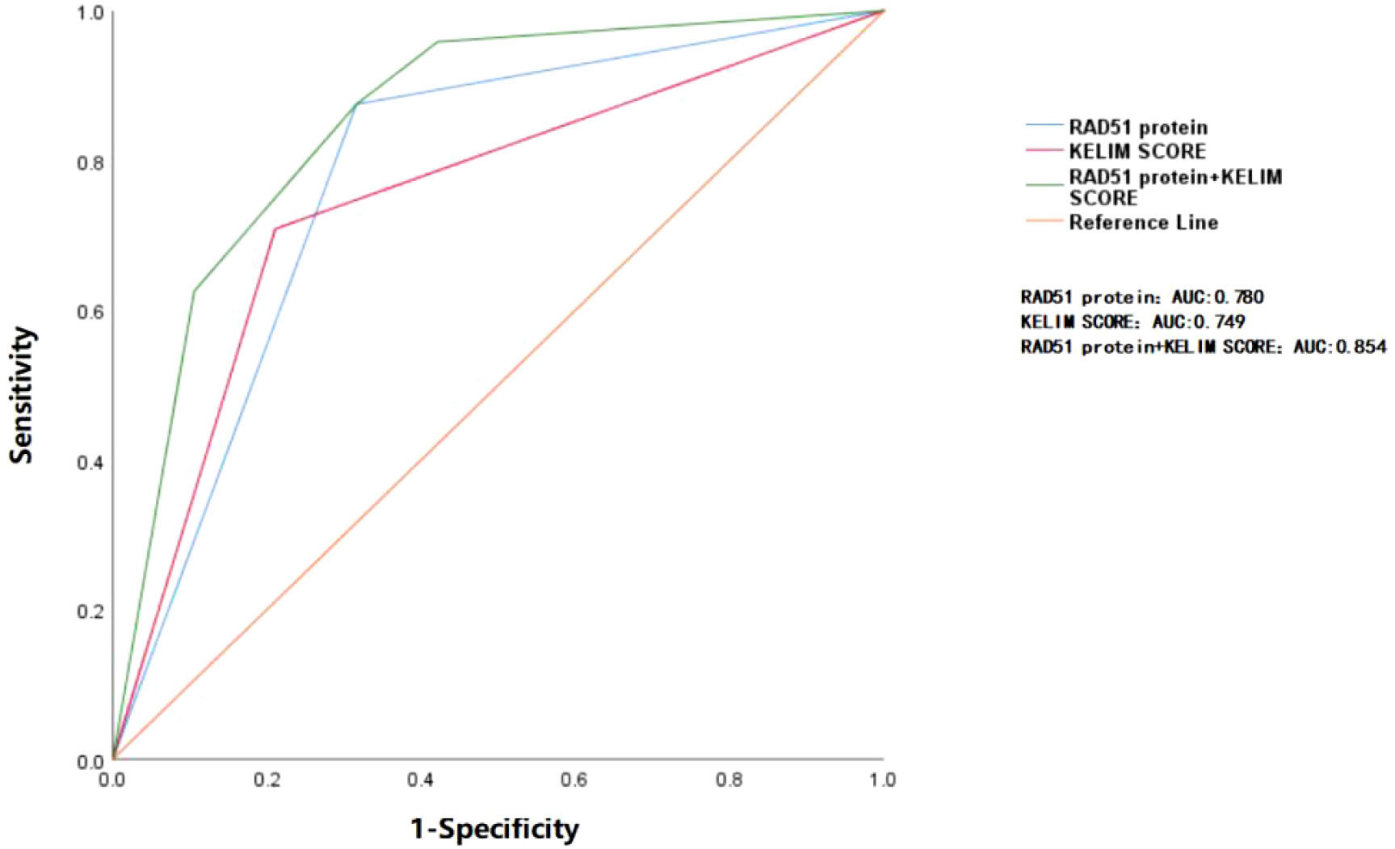
ROC curve analysis of RAD51 protein and KELIM score in predicting platinum resistance relapse. The area under the blue curve represents the AUC (0.780) for RAD51 protein alone in predicting the recurrence of platinum resistance. The area under the red curve corresponds to the AUC (0.749) for the KELIM score alone in predicting the same outcome. The area under the green curve reflects the AUC (0.854) when both RAD51 protein and KELIM score are used together for prediction. The differences between the three curves are not statistically significant (P > 0.05).

ROC curve analysis showed that the area under the curve (AUC) for predicting platinum resistance recurrence using RAD51 protein alone was 0.780, while the AUC for the KELIM score alone was 0.749. The difference between them was not statistically significant (P > 0.05). Although the AUC of the combination of RAD51 expression and the KELIM score showed an increasing trend (0.854) compared with the KELIM score and RAD51 expression respectively, the difference was not statistically significant (P > 0.05), as shown in **Figure 2**.

### 3.4 Expression trend of RAD51 protein before and after neoadjuvant chemotherapy and its relationship with platinum resistance relaps

In this study, we further analyzed the expression of RAD51 protein in tumor tissues from patients after interval debulking surgery (IDS). The results indicated that, among the 27 patients with low RAD51 expression before neoadjuvant chemotherapy (NACT), the RAD51 protein expression pattern ranged from low-to-high in 9/27 patients (33.3%) and remained low-to-low in 18/27 patients (66.7%). Among the 16 patients in the pre-NACT RAD51 high-expression group, 8/16 patients (50%) exhibited a high-to-low expression pattern, while 8/16 patients (50%) maintained high expression. After neoadjuvant chemotherapy, patients with a low-to-high RAD51 expression pattern had a significantly higher risk of platinum resistance relapse compared to those with a low-to-low expression pattern (P < 0.05). However, no significant change in the risk of platinum resistance relapse was observed in patients whose RAD51 expression shifted from high to low (P = 0.522). These findings are summarized in **Table 5**.

**Table 5:** Expression of RAD51 protein before and after NACT and its relationship with platinum resistance recurrence.

Variables	Platinum sensitivity or recurrence of platinum resistance			P
	≥ 6 n (%)	< 6 n (%)	χ^2^	
RAD51 expression				
Low-High	4 (19.0%)	5 (83.3%)	8.679	0.003
Low-low	17 (81.0%)	1 (16.7%)		
High - High	1 (33.3%)	7 (53.8%)	0.410	0.522
High -low	2 (66.7%)	6 (46.2%)		

### 3.5 PFS and OS survival analysis based on RAD51 protein expression

**Figure 3:**
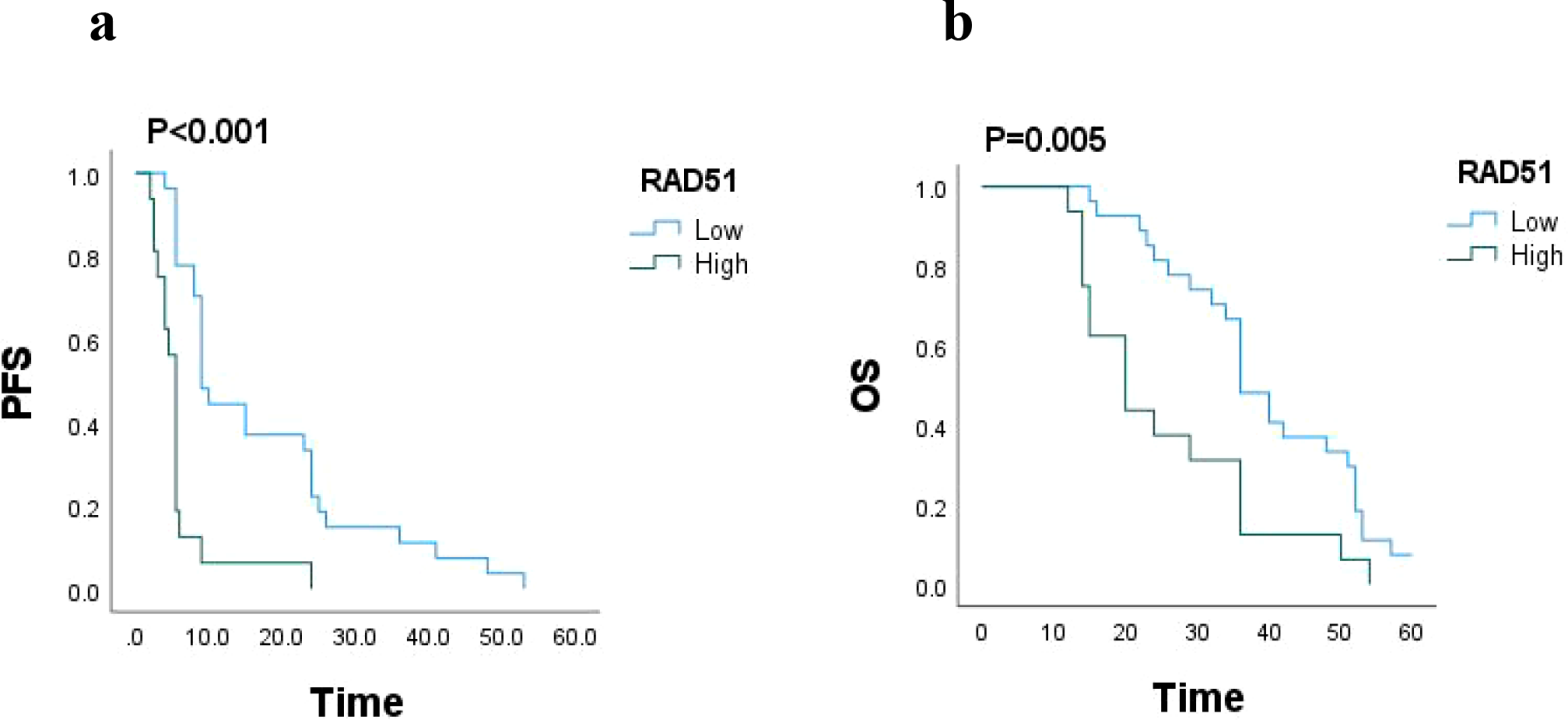
**(a)** PFS based on RAD51 protein expression (n = 43), P < 0.001. **(b)** OS based on RAD51 protein expression (n = 43), P = 0.005.

In this study, a total of 43 patients were followed up for a period of 5 years, with a mean follow-up duration of 30 months. Survival analysis was conducted to assess the impact of RAD51 protein expression on patient outcomes. The results revealed that the median progression-free survival (PFS) for patients with high RAD51 expression was 5.5 months (95% confidence interval [CI]: 4.990-6.010), while for those with low RAD51 expression, it was significantly longer, at 9 months (95% CI: 7.546-10.454; P < 0.001). Similarly, the median overall survival (OS) for patients with high RAD51 expression was 20 months (95% CI: 13.518-26.482), whereas for those with low RAD51 expression, it was 36 months (95% CI: 31.638-40.362; P = 0.005). These findings suggest that high RAD51 expression is associated with poorer PFS and OS, indicating a statistically significant worse survival prognosis for these patients. These results are visually represented in **Figure 3**.

## 4 Discussion

NACT has increasingly been used in the treatment of advanced epithelial ovarian cancer, with the aim of reducing tumor burden through initial chemotherapy followed by cytoreductive surgery. However, the cytotoxicity and mutations induced by chemotherapy can lead to tumor resistance (13). In patients with FIGO stage III and IV ovarian cancer, the combination of NACT and IDS (NACT-IDS) has been associated with increased resistance to platinum-based agents, a higher recurrence risk, and a decreased median overall survival (7, 14). Therefore, precise screening of patients suitable for NACT is crucial to minimize platinum resistance recurrence and improve survival outcomes.

RAD51, a key protein in DNA damage repair, catalyzes homologous recombination (HR) processes, facilitating homology recognition and strand exchange. Its overexpression enhances HR repair capacity, alters recombination pathways, increases genomic instability, and promotes carcinogenesis or cancer progression. RAD51 overexpression has been implicated in various cancers, including colon, breast, and ovarian cancers, and is strongly correlated with tumor progression and poor prognosis (15, 16). Platinum-based chemotherapy induces DNA double-strand breaks (DSBs), which is a primary mechanism of its cytotoxicity. High RAD51 expression facilitates rapid DNA repair, thereby conferring resistance to platinum-based chemotherapy and reducing chemosensitivity (17, 18).In this study, RAD51 expression was significantly higher in advanced high-grade serous ovarian carcinoma compared to normal ovarian tissue. Among patients with high RAD51 expression, 75.0% had a (12/16) KELIM score < 1, indicating poor chemotherapy sensitivity to NACT. Conversely, 63.0% of patients with low RAD51 expression had a KELIM score ≥ 1, indicating high chemosensitivity. These findings suggest that RAD51 expression in ovarian cancer tissues is closely associated with chemotherapy sensitivity to NACT.

Hoppe et al. (19) reported a strong correlation between elevated RAD51 expression and early recurrence following platinum-based therapy in ovarian cancer. They noted that tumors with high RAD51 expression were at a greater risk of developing primary platinum resistance. In this study, among patients who experienced platinum-resistant relapse, a subset had high RAD51 expression. Notably, another group of patients exhibited low RAD51 expression before neoadjuvant chemotherapy (NACT), but this expression transitioned to high levels after chemotherapy. These findings suggest that high RAD51 expression is not only associated with the recurrence of platinum resistance but also indicates that RAD51 expression is dynamic, changing before and after NACT. Similarly, Kim et al. (20) investigated RAD51 expression in tumor tissues from 34 patients with high-grade ovarian cancer (HGOC) before and after NACT. They found that 26.3% of patients in the pre-NACT low-RAD51 group exhibited high RAD51 expression post-chemotherapy. The mechanisms underlying the changes in RAD51 expression before and after NACT remain unclear. One possibility is that NACT-induced exogenous DNA damage triggers an increase in RAD51 expression, making tumor cells less responsive to chemotherapy and promoting the subsequent development of platinum resistance recurrence. Additionally, this study observed that even though some patients with high RAD51 expression transitioned to low expression after NACT, they still experienced recurrence of platinum resistance. These observations imply that high RAD51 expression, whether initially present or induced by NACT, confers significant biological activity to tumor cells. Further investigation into the regulatory mechanisms driving these changes is warranted.

Recent studies have indicated that the KELIM score is a valuable tool in assessing chemotherapy sensitivity and predicting platinum-based resistance recurrence and survival outcome (21–23). However, there are limitations to its use, particularly in predicting chemotherapy sensitivity prior to neoadjuvant chemotherapy (NACT) and identifying patients with primary drug resistance, as it requires multiple CA125 measurements after chemotherapy initiation. In the study mentioned, a strong correlation was found between high RAD51 expression and KELIM scores in pre-NACT tumor tissues and platinum resistance recurrence. Therefore, we further conducted the ROC curve analysis. The results showed that the RAD51 protein demonstrated the same predictive efficacy as the KELIM score in predicting the risk of platinum-resistant recurrence. Although the predictive efficacy of the combined application of the RAD51 protein and the KELIM score did not achieve the significant improvement as expected, which might be related to the relatively small sample size included in this study, further verification is needed by expanding the sample size and carrying out prospective studies. These research findings highlight the potential of RAD51 protein expression as a biomarker in evaluating the sensitivity of tumors to chemotherapy, identifying patients with primary platinum resistance, as well as its potential value in predicting chemosensitivity before neoadjuvant chemotherapy (NACT). By incorporating RAD51 expression into the assessment, clinicians can make more precise and rational decisions regarding the application of neoadjuvant chemotherapy.

Furthermore, the study by Feng et al. supports the significance of RAD51 expression in predicting survival outcomes. They found that patients with high RAD51 expression had poorer progression-free survival (PFS) and shorter overall survival (OS), indicating a reduced drug sensitivity and worse prognosis (24). Similarly, another study demonstrated the prognostic value of RAD51 expression in non-small cell lung cancer (NSCLC) patients undergoing neoadjuvant chemotherapy, further establishing RAD51 as a robust biomarker in this context (25). In the current study, a 5-year follow-up of 43 patients with HGOC revealed that those with high RAD51 expression had significantly lower median PFS and OS (5.5 vs 9; 20 vs 36). These results underscore that high RAD51 expression is linked to significantly worse survival outcomes, in line with previous findings.

In conclusion, this study demonstrates that RAD51 protein expression offers a valuable tool for predicting chemotherapy sensitivity, platinum resistance recurrence, and survival outcomes in patients with advanced epithelial ovarian cancer. Importantly, assessing RAD51 levels in tumor tissues provides crucial insights into chemotherapy responsiveness and helps predict patient prognosis, thus serving as a potential guide for making informed treatment decisions. These findings underscore the significance of RAD51 as a biomarker in ovarian cancer and highlight the need for further research to explore its clinical applications. However, several limitations should be noted. First, the sample size in this study is relatively small, and the analysis of patient prognosis is constrained; hence, further validation in a larger cohort is warranted. Additionally, the study exclusively focused on patients who underwent successful tumor cytoreductive surgery (at least R1) after IDS. For those who did not achieve optimal cytoreduction, the role of RAD51 expression in patient prognosis requires further investigation in a multicenter, large-sample prospective study. Future research should focus on the development of targeted therapies targeting RAD51, with the goal of enhancing chemotherapy sensitivity by down-regulating RAD51 expression, ultimately improving survival outcomes for patients with ovarian cancer.

## Data Availability

The original contributions presented in the study are included in the article/supplementary material. Further inquiries can be directed to the corresponding author.
